# Psychological impact of lymphoma on adolescents and young adults: not a matter of black or white

**DOI:** 10.1007/s11764-016-0518-7

**Published:** 2016-02-08

**Authors:** F. M. Drost, F. Mols, S. E. J. Kaal, W. B. C. Stevens, W. T. A. van der Graaf, J. B. Prins, O. Husson

**Affiliations:** CoRPS - Centre of Research on Psychology in Somatic Diseases, Department of Medical and Clinical Psychology, Tilburg University, Tilburg, The Netherlands; Comprehensive Cancer Centre the Netherlands (CCCN), Netherlands Cancer Registry, Eindhoven, The Netherlands; Department of Medical Oncology, Radboud University Medical Centre, Nijmegen, The Netherlands; Department of Haematology, Radboud University Medical Centre, Nijmegen, The Netherlands; The Institute of Cancer Research and Royal Marsden NHS Foundation Trust, London, UK; Department of Medical Psychology, Radboud University Medical Centre, PO Box 9101, 6500 HB Nijmegen, The Netherlands

**Keywords:** Adolescent and young adult oncology, Impact of cancer, Lymphoma, Health-related quality of life

## Abstract

**Purpose:**

The purpose of the study is to examine differences in perceived impact of cancer (IOC) between adolescents and young adults (AYAs; 18–35 years at cancer diagnosis), adults (36–64 years) and elderly (65–84 years) with a history of (non-)Hodgkin lymphoma. Furthermore, to investigate the association of socio-demographic, clinical and psychological characteristics with IOC; and the association between IOC and health-related quality of life (HRQoL) among AYAs only.

**Methods:**

This study is part of a population-based PROFILES registry survey among lymphoma patients diagnosed between 1999 and 2009. Patients (*n* = 1.281) were invited to complete the IOCv1 and EORTC-QLQ-C30 questionnaires. Response rate was 67 % (*n* = 861).

**Results:**

AYA lymphoma survivors scored higher on the positive IOC summary scale, compared to adult and elderly patients (*p* < 0.001), while no significant differences were observed for negative IOC. Among AYAs, females, survivors with a partner, and survivors with elevated psychological distress levels scored significantly higher on the negative IOC summary scale. The negative IOC summary scale was negatively associated with all EORTC QLQ-C30 functioning scales (β ranging from −0.39 to −0.063; *p* < 0.05). The positive IOC summary scale was negatively associated with the EORTC QLQ-C30 subscale ‘*Emotional functioning*’ (β = −0.24; *p* < 0.05).

**Conclusion:**

AYA, adult and elderly with a history of (non-)Hodgkin lymphoma experienced different types of IOC in terms of positive and negative aspects.

**Implications for Cancer Survivors:**

Although AYAs experience a more positive IOC compared to older survivors, some AYAs experience more negative IOC and may require developmentally appropriate interventions to address their specific concerns.

## Introduction

In The Netherlands, each year approximately 4000 people are diagnosed with non-Hodgkin lymphoma (NHL) and 400 with Hodgkin lymphoma (HL) [1]. Advances in early cancer detection, diagnosis, and treatments have noticeably improved survival rates of patients with lymphoma [2–4]. The 5-year survival rates range from 48–76 % (NHL) to 95 % (HL), depending on type and stage of tumour, treatment, and age of the patient [1]. In 2013, there were approximately 5800 patients living with a history of HL and 30,000 with NHL [1].

As the survival rate is increasing, attention is more and more given to the long-term consequences (e.g. fatigue, depression) and late effects (e.g. infertility and second cancers) of cancer and its treatment. These effects can have a negative influence on health-related quality of life (HRQoL) [5–8]. In addition, cancer survivors also perceive specific positive and negative impact of cancer (IOC) on their lives. In a positive way, cancer survivors can develop more empathy and health awareness, or a positive self-evaluation after their cancer [9]. On the other hand, cancer can influence life in a negative way, by appearance and body change concerns, and by worrying [9]. Development of the IOC questionnaire was spurred by the need to measure unique aspects of survivorship not addressed by existing HRQoL measures [9]. Whereas HRQoL measures generally focus on physical, cognitive, social and emotional functioning, the IOC measure addresses the unique issues, problems and changes that long-term survivors ascribe to their cancer experience (e.g. the survivors ‘*health awareness*’, the ‘*meaning of cancer*’ and how the experience changed the survivor) [9].

IOC may be age-specific [5, 10, 11]. Each phase in life is characterized by developmental milestones or typical life concerns [12]. For example, elderly people (>65 years at diagnosis) generally have to deal with a decline in physical functioning, less social support, isolation and comorbid conditions [13–15]. In contrast, adults (36–64 years at diagnosis) more often have concerns about unemployment, financial issues and family matters [16, 17], while adolescents and young adults (AYAs; 18–35 years at cancer diagnosis) are in a challenging period where they face major life tasks, such as establishing their personal identity, creating intimate relationships, gaining independence and starting careers and families [18–20]. A cancer diagnosis at AYA age can significantly disrupt or delay achieving these milestones [21]. Understanding age-related differences in IOC can contribute to the development of age-appropriate psychological interventions and personalized care. Therefore, the primary aim of the present study was to compare the IOC between AYA, adult and elderly cancer survivors. Other study objectives were to (1) investigate the influence of AYA characteristics on IOC and (2) examine the association between IOC and HRQoL among AYAs.

## Methods

### Setting and population

This study is part of a longitudinal population-based survey among lymphoma survivors registered within the Netherlands Cancer Registry (NCR) in the South of The Netherlands. The NCR records data of all patients newly diagnosed with cancer in the southern part of The Netherlands, an area with 2.3 million inhabitants. The NCR was used to select all patients diagnosed with NHL and HL between January 1, 1999 and January 1, 2009. We included patients aged ≥18 years at time of diagnosis, with all subtypes of indolent (including chronic lymphocytic leukaemia-like) and aggressive B cell NHL and HL as defined by the International Classification of Diseases for Oncology-3 codes (ICD-O-3) [22]. Deceased patients were excluded by linking the NCR database with the Central Bureau for Genealogy. Ethical approval for the study was obtained from the local certified Medical Ethics Committee of the Maxima Medical Centre Veldhoven.

### Data collection

Data collection took place in 2009 and was done within PROFILES [23]. PROFILES is a registry for the study of the physical and psychosocial impact of cancer and its treatment from a dynamic, growing population-based cohort of both short- and long-term cancer survivors. PROFILES is linked directly to clinical data from NCR. Details of the data collection method have been previously described [23]. Data from the PROFILES registry are available for non-commercial scientific research, subject to study question, privacy and confidentiality restrictions, and registration (www.profilesregistry.nl).

In May 2009, patients between 6 months and 10 years after diagnosis were included in the study. In November 2009, patients diagnosed between May and August 2009 were also invited to participate to broaden the number of short-term survivors.

### Study measures

#### Sociodemographic and clinical characteristics

Clinical information was available from the NCR that routinely collects data on tumour characteristics, primary treatment and patients’ background characteristics including sex and date of birth. Comorbidity at time of survey was assessed using a modified version of the Self-Administered Comorbidity Questionnaire assessing the prevalence of 14 comorbidities including heart disease, stroke, high blood pressure, COPD/asthma, diabetes, stomach disease, kidney disease, liver disease, anaemia, depression, thyroid disease, osteoarthritis, back pain, and rheumatoid arthritis [24]. Self-designed questions on educational level and marital status were added to the questionnaire.

### Impact of cancer

The 41-item IOCv1 was used in this study to measure specific positive and negative IOC among long-term cancer survivors [9, 25]. Patients could indicate their level of agreement on each statement from 1 (*strongly disagree*) to 5 (*strongly agree*). The questionnaire was missing one item of the ‘negative self-evaluation’ subscale by mistake. This missing item was imputed by the mean score of the ‘negative self-evaluation’ subscale for each participant separately. A more recent and comprehensive scaling of the IOCv1 questionnaire yielded the 37-item IOCv2 [26]. A valid algorithm was used to convert the IOCv1 item responses into IOCv2 item scores [27]. Two main summary IOC scales could be formed, a positive (*α* = 0.74) and a negative (*α* = 0.82). The total scale scores were calculated by averaging the item scores. Higher scores on the positive and negative impact summary scales indicate greater positive and negative impacts of cancer, respectively. The two summary scales could be further subdivided into eight subscales. The four positive subscales comprise: *altruism/empathy*, *health awareness*, *meaning of cancer* and *positive self-evaluation*. The negative subscales are *appearance concerns*, *body change concerns*, *life interferences* and *worry*.

### Health-related quality of life

The validated Dutch version of the EORTC QLQ-C30 was used to assess HRQoL. The questionnaire includes five scales on physical (*α* = 0.79), role (*α* = 0.89), emotional (*α* = 0.90), cognitive (*α* = 0.73) and social functioning (*α* = 0.79); and a global health status/quality of life scale (*α* = 0.92). Answer categories range from 1 (not at all) to 4 (very much). After linear transformation, all scales range in score from 0 to 100. A higher score means a better HRQoL [28].

### Statistical analysis

Analyses were performed using Statistical Package for the Social Sciences version 22 (SPSS), Chicago, IL, USA and two-sided *p* values of <0.05 were considered statistically significant. Differences in demographic and clinical characteristics between respondents, non-respondents and cancer survivors with unverifiable addresses and between the three age groups (AYA, adults, elderly) were compared using chi-square and analysis of variance (ANOVA), where appropriate.

ANOVAs were performed to investigate mean differences between the age categories of lymphoma survivors (independent variable) and the IOCv2 total and subscale scores (dependent variables). To counteract the problem of multiple comparisons (type-1 error), Bonferroni correction was used (*p* < 0.001).

Multiple linear regression analyses were used to compare AYA lymphoma survivor characteristics (independent variables) and the mean IOCv2 summary and subscale scores (dependent variables).

Additionally, multiple linear regression analyses were performed to investigate the independent association between IOCv2 total and subscales, and the HRQoL subscales for the AYA sample. All demographic and clinical variables were included as confounders; this was determined a priori based on hypotheses.

## Results

### Clinical and sociodemographic characteristics

Of the 1281 Dutch lymphoma survivors who met the eligibility criteria and received a questionnaire, 861 (67 % response rate) completed it.

A comparison of respondents, non-respondents and survivors with unverifiable addresses, indicated that non-respondents were older, more recently diagnosed or less often diagnosed with stage I disease, when compared to survivors with unverifiable addresses and respondents.

Of all respondents, 11 % was AYA (18–35 years at diagnosis), 59 % adult (36–64 years at diagnosis) and 30 % elderly (65–84 years at diagnosis).

AYA survivors were more often diagnosed with HL and more often with stage II disease and treated more often with a combination of chemotherapy and radiotherapy, when compared to older lymphoma survivors. Additionally, AYAs reported fewer comorbid conditions, were married less often, and more often had a higher educational level and a job compared to adult and elderly lymphoma survivors (Table 1).

**Table 1:** Sociodemographic and clinical characteristics of cancer survivors stratified by age

	AYA	Adults	Elderly	*p* value
	*N* = 98	*N* = 507	*N* = 256	
Age (at time of diagnosis) (mean ± SD)	28.0 (5.4)	53.4 (8.0)	71.4 (4.0)	<0.01
Age (at time of survey) (mean ± SD)	32.9 (6.0)	58.5 (8.2)	75.7 (4.0)	<0.01
Tumour type				<0.01
NHL	33 (34 %)	435 (86 %)	245 (96 %)	
HL	65 (66 %)	71 (14 %)	10 (4 %)	
Years since diagnosis (mean ± SD)	5.0 (2.5)	5.1 (2.6)	4.3 (2.4)	<0.01
Years since diagnosis				<0.01
<2 years	12 (12 %)	68 (14 %)	45 (18 %)	
2–5 years	39 (40 %)	191 (38 %)	128 (51 %)	
5–10 years	47 (48 %)	233 (46 %)	76 (30 %)	
> 10 years	0 (0 %)	11 (2 %)	4 (2 %)	
Sex				0.08
Male	49 (50 %)	306 (60 %)	161 (63 %)	
Female	49 (50 %)	200 (40 %)	94 (37 %)	
Stage at diagnosis				<0.01
I	19 (19 %)	120 (24 %)	64 (25 %)	
II	49 (50 %)	92 (18 %)	42 (17 %)	
III	19 (19 %)	71 (14 %)	30 (12 %)	
IV	10 (10 %)	121 (24 %)	59 (23 %)	
Unknown	1 (1 %)	102 (20 %)	60 (24 %)	
Primary treatment				<0.01
Radiotherapy alone	5 (5 %)	43 (9 %)	23 (9 %)	
Chemotherapy alone	38 (39 %)	227 (46 %)	125 (50 %)	
Chemotherapy + radiotherapy	54 (55 %)	98 (20 %)	32 (13 %)	
Active surveillance^a^	0 (0 %)	121 (24 %)	66 (26 %)	
Surgery	1 (0.5 %)	5 (0.7 %)	0 (0 %)	
Transplantation	0 (0 %)	1 (0.2 %)	0 (0 %)	
Other therapies	1 (1 %)	4 (1 %)	6 (2 %)	
Comorbidity				<0.01
None	63 (64 %)	187 (37 %)	65 (26 %)	
1	21 (21 %)	154 (30 %)	70 (28 %)	
2	8 (8 %)	83 (16 %)	63 (25 %)	
3 or more	6 (6 %)	82 (16 %)	57 (22 %)	
Marital status				<0.01
Married/cohabiting	66 (68 %)	426 (85 %)	181 (74 %)	
Divorced/separated	11 (11 %)	29 (6 %)	12 (5 %)	
Widowed	1 (1 %)	28 (6 %)	42 (17 %)	
Never married/never cohabiting	19 (20 %)	19 (4 %)	11 (5 %)	
Education level				<0.01
Low	1 (1 %)	57 (11 %)	64 (26 %)	
Medium	54 (57 %)	314 (63 %)	138 (56 %)	
High	40 (42 %)	129 (26 %)	43 (18 %)	
Current occupation				<0.01
Employed	64 (74 %)	173 (37 %)	2 (1 %)	
Not working/retired	22 (26 %)	296 (63 %)	244 (99 %)	

*AYA* (18–35 years), *Adults* (36–64 years), *Elderly* (65–84 years): age at time of diagnosis

*NHL* non-Hodgkin lymphoma, *HL* Hodgkin lymphoma education levels included *low* no/primary school, *medium* lower general secondary education/vocational training, or high pre-university education/high vocational training/university

^a^Patients under active surveillance and receive no therapy

### Associations between age and IOC

Significant differences in IOC between the age groups were observed on the positive impact summary scale (F(2798) = 11.54, *p* < 0.001, eta^2^ = 0.03) and on the positive subscales ‘health awareness’ (*p* < 0.001), ‘meaning of cancer’ (*p* = 0.001) and ‘positive self-evaluation’(*p* < 0.001). No significant differences between the age groups were found on negative IOC (Table 2). Sub analyses for HL survivors only showed the same mean scale scores only statistical significance of these findings could not be determined due to the small number of elderly with HL (*n* = 10; data not shown).

**Table 2:** Mean scores of the Impact of Cancer (IOCv2) sub and total scales (±SD) according to age

	AYA	Adults	Elderly	*p* value
	*N* = 98	*N =* 507	*N =* 256	
IOCv2	Mean (SD)	Mean (SD)	Mean (SD)	
Altruism/empathy	3.3 (0.7)	3.2 (0.8)	3.2 (0.9)	0.64
Health awareness	3.5 (0.9)	3.3 (0.8)	3.0 (1.0)	<0.001ª
Meaning of cancer	2.8 (0.8)	2.7 (0.8)	2.5 (0.9)	0.001ª
Positive self-evaluation	3.8 (0.7)	3.4 (0.8)	3.2 (0.9)	<0.001^b^
Appearance concerns	1.8 (1.0)	1.8 (0.8)	1.8 (0.8)	0.94
Body change concerns	2.6 (1.1)	2.6 (1.0)	2.6 (1.0)	0.51
Life interferences	1.9 (0.5)	2.0 (0.5)	2.0 (0.5)	0.09
Worry	2.6 (0.9)	2.8 (0.9)	2.6 (1.0)	0.005
Positive impact scale	3.4 (0.6)	3.1 (0.6)	3.0 (0.7)	<0.001^c^
Negative impact scale	2.2 (0.7)	2.3 (0.7)	2.2 (0.7)	0.19

Bonferonni correction *p* = 0.01/10 = <0.001

*IOCv2* Impact of Cancer Scale version 2: high scores representing greater impact

*AYA* (18–35 years), *Adults* (36–64 years), *Elderly* (65–84 years)

^a^Between AYA and elderly and between adults and elderly

^b^Between AYA and adults and between AYA and elderly

^c^Between AYA and adults, between AYA and elderly and between adults and elderly

### Associations between AYA characteristics and IOC

Female AYAs scored significantly higher on the negative impact summary scale and on the subscales ‘appearance concerns’ and ‘positive self-evaluation’ compared to AYA males. AYA survivors with a partner scored higher on the negative impact summary scale and ‘body change concerns’. AYAs with more comorbidities reported more impact on ‘appearance concerns’ and AYA survivors with elevated psychological distress scored significantly higher on all IOCv2 scales, except for the positive impact summary scale and the subscales ‘meaning of cancer’ and ‘positive self-evaluation’ (Table 3).

**Table 3:** Multiple linear regression analyses of IOCv2 total and subscales of AYA lymphoma survivors (*N* = 98)

	Positive subscales					Negative subscales				
	Positive impact	Altruism/empathy	Health awareness	Meaning of cancer	Positive self-evaluation	Negative impact	Appearance concerns	Body change concerns	Life interferences	Worry
	Bèta	Bèta	Bèta	Bèta	Bèta	Bèta	Bèta	Bèta	Bèta	Bèta
Age (at time of survey)	0.05	0.04	0.13	−0.02	−0.08	0.00	0.06	−0.04	−0.04	0.11
Tumour type										
NHL	Ref	Ref	Ref	Ref	Ref	Ref	Ref	Ref	Ref	Ref
HL	−0.03	−0.06	0.10	−0.10	0.06	−0.13	−0.17	−0.03	−0.11	−0.02
Years since diagnosis										
≤5 years	Ref	Ref	Ref	Ref	Ref	Ref	Ref	Ref	Ref	Ref
>5 years	0.14	0.19	0.04	0.10	0.03	−0.01	−0.19	0.08	−0.06	0.03
Sex										
Male	Ref	Ref	Ref	Ref	Ref	Ref	Ref	Ref	Ref	Ref
Female	0.10	−0.10	0.21	−0.03	0.30*	0.22*	0.21*	0.13	0.15	0.11
Education										
Low	−0.01	0.11	0.05	−0.14	−0.06	0.04	−0.06	0.04	−0.01	0.10
Medium	−0.02	−0.12	−0.00	−0.13	0.18	0.14	0.14	0.18	0.11	0.08
High	Ref	Ref	Ref	Ref	Ref	Ref	Ref	Ref	Ref	Ref
Current occupation										
Employed	Ref	Ref	Ref	Ref	Ref	Ref	Ref	Ref	Ref	Ref
Not working/retired	−0.06	−0.01	−0.11	0.17	−0.25	0.09	0.16	0.10	0.12	−0.04
Marital status										
No partner	Ref	Ref	Ref	Ref	Ref	Ref	Ref	Ref	Ref	Ref
Partner	0.05	0.10	0.11	−0.07	−0.03	0.22*	0.15	0.24*	0.16	0.14
Comorbidity										
No	Ref	Ref	Ref	Ref	Ref	Ref	Ref	Ref	Ref	Ref
Yes	0.07	0.03	0.02	0.01	0.08	0.15	0.24*	0.17	0.08	0.06
Psychological distress (HADS)										
≤15	Ref	Ref	Ref	Ref	Ref	Ref	Ref	Ref	Ref	Ref
>15	0.24	0.28*	0.29*	0.00	0.12	0.44**	0.31**	0.41**	0.49**	0.31**

*NHL* non-Hodgkin lymphoma, *HL* Hodgkin lymphoma; Education levels included *low* = no/primary school, *medium* = lower general secondary education/vocational training, or *high* = pre-university education/high vocational training/university

*HADS* Hospital Anxiety Depression Scale

*p < 0.05; **p < 0.01

### Associations between IOC and HRQoL among AYA lymphoma survivors

After adjusting for sociodemographic and clinical factors, the negative impact summary scale was negatively associated with all HRQoL scales (Table 4). The positive impact summary scale was negatively associated with the EORTC QLQ-C30 subscale ‘Emotional functioning’, which was mainly caused by the items of the IOC subscales ‘altruism/empathy’ and ‘health awareness’.

**Table 4:** Multiple linear regression analyses of the association between IOCv2 total and subscales and HRQOL (EORTC QLQ-C30) subscales for the AYA lymphoma survivors

	Physical functioning		Role functioning		Emotional functioning		Cognitive functioning		Social functioning		Global health status	
	Bèta		Bèta		Bèta		Bèta		Bèta		Bèta	
EORTC QLQ-C30	Unadjusted	Adjusted	Unadjusted	Adjusted	Unadjusted	Adjusted	Unadjusted	Adjusted	Unadjusted	Adjusted	Unadjusted	Adjusted
Positive impact	−0.10	−0.06	0.03	0.05	−0.27**	−0.24*	−0.18	−0.14	−0.09	−0.06	−0.16	−0.13
Altruism/empathy		−0.01		0.04		−0.30**		−0.21*		−0.06		−0.25*
Health awareness		−0.17		−0.13		−0.31**		−0.30**		−0.25*		−0.21
Meaning of cancer		−0.06		0.05		−0.16		0.01		0.06		−0.02
Positive self-evaluation		0.04		0.19		−0.11		0.08		0.08		0.09
Negative impact	−0.56**	−0.44**	−0.50**	−0.39**	−0.55**	−0.59**	−0.67**	−0.63**	−0.51**	−0.54**	−0.64**	−0.60**
Appearance concerns		−0.32**		−0.16		−0.39**		−0.35**		−0.15		−0.37**
Body change concerns		−0.49**		−0.38**		−0.55**		−0.58**		−0.50**		−0.59**
Life interferences		−0.42**		−0.39**		−0.57**		−0.64**		−0.61**		−0.59**
Worry		−0.29**		−0.29*		−0.42**		−0.37*		−0.31**		−0.39*

Adjusted for age at time of survey, tumour type, years since diagnosis, sex, education, current occupation, marital status, comorbidity

*EORTC QLQ C-30* European Organization for Research and Treatment of Cancer Quality of Life Core questionnaire, *IOCv2* Impact of Cancer Scale version 2

*p < 0.05; **p < 0.01

## Discussion

This study showed that AYA, adult and elderly (non-)Hodgkin lymphoma survivors experienced different types of positive and negative IOC. Our results are in line with those of previous studies among lymphoma survivors, which showed that younger survivors had higher positive impact scores compared to older survivors [11, 29]. The positive IOC among AYAs is reflected in more ‘*health awareness’* (e.g. better self-care), ‘*meaning of cancer’* (e.g. giving direction in life) and ‘*positive self-evaluation’* (e.g. considering oneself to be a role model). An explanation could be that AYAs make use of more active and adaptive coping mechanisms and might be more resilient compared to older cancer survivors [30–33]. Resilience is described as a personality profile characterized by ‘positive adaptation’ after a stressful life event [34, 35]. Moreover, studies show that younger cancer survivors report higher levels of post-traumatic growth [36, 37], which refers to a set of positive changes occurring as a result of coping with a traumatic event like cancer. AYAs might experience more personal growth after their cancer experience compared to older cancer survivors as they are more likely to give direction in life and report adequate health competence after a cancer experience [38]. On the other hand, contrary to cancer survivors, healthy older adults are more resilient than young adults [39].

In contrast to previous studies [5, 11, 29], showing that younger cancer survivors had higher negative impact scores when compared to older survivors, our results showed no significant differences for negative impacts of cancer between the three age groups. One possible explanation could be that the majority of lymphoma survivors did not undergo invasive or surgical treatments and they experienced, for example, less ‘*body change concerns’* or *‘appearance concerns’* compared to survivors treated for other types of cancer. Another explanation could be that the IOC questionnaire lacks important age-specific concerns. Development of more age-specific questionnaires will make it possible to accurately identify IOC and prioritize the perceived age-specific needs of cancer survivors [40, 41].

Female AYAs scored significantly higher on the negative IOC, compared to young men. This gender difference is largely consistent with previous studies on long-term cancer survivors of all ages [5, 42]. A possible explanation could be the presence of higher rates of psychological distress in the female population in general, as opposed to men [43, 44]. This sex difference may reflect a difference in willingness to report distress among men, but could also occur because women tend to use more emotional coping styles [45–47]. The negative IOC among female AYAs is reflected in more ‘*appearance concerns*’ compared to men. This is in line with the literature which shows that especially young women, report more experiences associated with cancer-related appearance and body image concerns, than young men cancer survivors [48]. For example, women are more aware of their looks, and may feel more disfigured than men. On the other hand, female AYAs reported higher scores on the positive subscale ‘*positive self-evaluation*’. This means that young women consider themselves as a cancer survivor and/or role model, and may feel a sense of pride from surviving cancer. A possible explanation for this finding could be the tendency of women to score higher on post-traumatic growth. This explanation suggests that men and women differ in their responses to a traumatic (cancer) experience [49]. Moreover, an American study found that the post-traumatic growth scores of severely traumatized women were twice as high as those of traumatized men. It suggests that these events have greater effect on women, in that women may be more capable than men of learning or benefiting from difficult life experiences like surviving cancer [49].

The majority of AYA cancer survivors in this study reported that having more psychological distress in general life results in higher scores on the negative IOC summary scale. This was reflected in more ‘*appearance concerns’*, ‘*body change concerns’*, ‘*life interferences’* and ‘*worry’*. On the other hand, more psychological distress had a more positive impact on the positive subscales ‘*altruism/empathy*’ and ‘*health awareness*’. One possible explanation for this conflicting finding could be that cultural differences between lymphoma survivors living in the USA (where the IOC questionnaire was developed) and Dutch lymphoma survivors affected the IOC scores [11]. Living in different cultures creates other psychological resources that influence health. Since the sense of personal control is more prevalent in North America than in Europe, American survivors are more likely to alter perceptions of the cancer experience in a more positive way [50]. To give an example, the subscale ‘*health awareness*’ is seen as something positive in the USA, while in The Netherlands, we may experience it as something more negative [11]. Moreover, the conflicting finding that AYAs with elevated levels of distress experience both negative and positive IOC may indicate the coexistence of the unique negative and positive influences of cancer further in life. This gives reason to belief that mental health is not a single continuum with negative impacts (distress) on one, and positive impacts (growth) on the other side, but rather represents a bivariate construct with two separate dimensions [51].

AYA cancer survivors with a partner scored significantly higher on the negative IOC summary scale and the subscale ‘*body change concerns*’, compared to AYAs without a partner. This finding seems very age-related, as previous studies on the general cancer population of all ages show that having a partner reduces the negative impacts of cancer [5, 21]. A first possible explanation could be that AYAs are in a vulnerable phase of life, in which intimate and sexual relationships are evolving. Especially cancer residual symptoms like ‘*body change concerns*’ and altered body image could have a more negative impact on AYAs with a partner when compared to AYAs without a partner [52]. Feelings of being unattractive, different or imperfect, or possible impairments in social skills could be barriers for a romantic relationship and may lead to more negative impacts of cancer [53]. Second, AYAs are in a phase of life in which plans for having children may arise. Especially AYAs with a partner may worry about infertility concerns after their cancer treatment, more than AYAs without a partner. Previous research shows that more than fifty percent of AYAs with a partner reported that their cancer experience had a negative impact on their plans for having children [21]. Third, AYA cancer survivors with a partner may experience feelings of guilt and shortcoming, because they “dragged” their partner into a medical situation with an uncertain future. Discordant feelings between the AYA cancer survivors and their partner could arise and have a negative impact on the survivors life [54]. These factors may contribute to higher negative IOC scores among AYAs with a partner.

Consistent with other studies [25], our findings indicate that an individual’s perception of the positive and negative IOC on various life domains is related to their functional and global health status. Especially the negative IOC is of influence and is significantly associated with HRQoL. These findings provide evidence for the notion that negative experiences may be more heavily related to overall well-being and functioning than positive experiences of cancer among AYAs. A possible explanation could be the fact that perceived negative outcomes may be more heavily weighted in determining psychological adjustment than positive change [55]. Additionally, as female AYAs, AYAs with elevated levels of distress, and AYAs with a partner reported higher levels of negative IOC, they are considered a “high-risk” population of having a lower HRQoL. Especially these groups of AYA lymphoma survivors should be screened for their HRQoL and should be more intensively guided by health care practitioners.

This study has a few limitations. First, although information was present concerning demographic and clinical characteristics of the non-respondents and survivors with unverifiable addresses, it remains unknown why non-respondents declined to participate in the study. Secondly, it cannot be ruled out that part of the differences in IOC subscale scores between the age groups are a result of the differences in tumour types diagnosed (and accompanied treatment regimens) between younger and older patients. Older patients more often have indolent NHL with a wait and see policy (and therefore probably more late effects of the cancer itself), while younger patients more often have aggressive NHL or HL and therefore receive a combination of chemo- and radiotherapy. However, sub analyses for HL survivors only showed the same mean IOC scale scores as for the total lymphoma sample, indicating that our results are mainly caused by age. Third, the cross-sectional design of our study limits the determination of causal associations between the study variables. Fourth, although the IOCv1 and v2 scales showed good psychometric properties among the whole age range, both do not include domains related to developmental issues (e.g. lagging behind healthy peers; difficulties to return to school or problems with getting a job) which are particularly important for AYAs. Fifth, no data were available of healthy AYAs as the IOC is a cancer-specific measure. The question remains whether young people in general experience more positive outcomes than older people or that our results indicate a cancer-specific effect of age [39]. Sixth, the study results cannot be generalized for the total AYA population as Hodgkin’s disease has a relatively good prognosis, what may give a more positive indication, compared to other types of cancer.

To promote the positive IOC among AYAs, future research in the area of cognitive and behavioural interventions (e.g. cognitive re-framing) is needed to determine if positive reinterpretation of a negative traumatic event could subsequently lead to adequate resilience skills and coping mechanisms, more positive growth, improved HRQoL and thereby possibly indirectly diminishing the negative IOC. For example, to improve the coping mechanisms of AYAs, psychological interventions directed at positive reframing of cancer should see the cancer experience as an opportunity to negotiate new challenges, re-evaluate life goals and priorities, and enhance self-knowledge may be particularly relevant and effective, especially for younger survivors. Additionally, recommended psychosocial intervention programs could include among others positive peer support, as well as psychoeducational interventions and therapies that focus on engaging cancer patients and facilitating change, by encouraging patients’ flexibility to accept what cannot be altered and committing themselves to what can be achieved (e.g. ‘Acceptance and Commitment Therapy’) [56–58]. On the other hand, to diminish or prevent negative IOC, health care providers may offer age-appropriate information to AYAs [56, 59], which may help them to get a more coherent understanding of their illness [51, 60, 61].

To conclude, this study highlights the importance of the specific IOC of AYAs according to their life phase, as compared to adults and the elderly lymphoma survivors. To address the unique needs of AYAs, research should focus on developing questionnaires and interventions more coherent to the age of the cancer survivor. Randomized intervention studies with large samples that focus on psychosocial outcomes are needed to establish evidence-based psycho-oncological interventions for AYAs. Healthcare practitioners need to become aware of the specific age-related impacts of cancer and developmental issues that AYA, adult and elderly lymphoma survivors are dealing with, even years after diagnosis. By keeping this in mind, healthcare practitioners can contribute to a better HRQoL and a more adaptive and supportive healthcare system for the lymphoma survivors.
